# 65. Impact of an Antibiotic Side-Chain-Based Cross-Reactivity Chart on Antibiotic Use in Patients With β-lactam Allergies and Pneumonia

**DOI:** 10.1093/ofid/ofab466.267

**Published:** 2021-12-04

**Authors:** Curtis D Collins, Nina West, Tara Shankar, Harvey L Leo, Renee Bookal

**Affiliations:** 1 St. Joseph Mercy Health System, Ann Arbor, Ypsilanti, Michigan; 2 St. Joseph Mercy Health System, Ypsilanti, Michigan; 3 Allergy and Immunology Associates of Ann Arbor, Ann Arbor, Michigan

## Abstract

**Background:**

β-lactam antibiotics with dissimilar R-group side chains are associated with low cross-reactivity. Despite this, patients with β-lactam allergies are too often treated with alternative antibiotic therapy. An institutional β-lactam side-chain-based cross-reactivity chart was developed and implemented to guide in antibiotic selection for β-lactam allergies patients.

**Methods:**

This single center, retrospective, cohort study analyzed the impact of the implementation of the cross-reactivity chart for patients with documented β-lactam allergies with pneumonia. Study time periods were defined as January 2013 to October 2014 prior to implementation of the chart (historical cohort) and January 2017 to October 2018 (intervention cohort) following institutional implementation and adoption. The primary outcome was the incidence of β-lactam utilization between time periods. Propensity-weighted scoring and interrupted time-series analyses compared outcomes across time periods.

**Results:**

A total of 341 and 623 patient encounters were included in the historical and intervention cohorts, respectively. There was a significant increase in the use of β-lactams for treatment of pneumonia (70.4% vs 89.3%; p < 0.001) and the use of any alternative therapy decreased between cohorts (58.1% vs. 36%; p < 0.001) (Figure 1). β-lactam use per patient significantly improved between cohorts in patients with mild, Type 1 IgE-mediated hypersensitivity reactions (HSRs) and in patients with unknown reactions. There was no difference in overall HSRs between cohorts (2.4% vs. 1.45; p = 0.628), or in patients who received β-lactam antibiotics (1.3% historical group vs 1.1% intervention group; p = 0.467). Median alternative antibiotic days of therapy (3 vs. 2; p = 0.027) and duration of therapy per patient (3 days vs. 2 days; p = 0.023) decreased between cohorts. There was a significant increase in mortality while healthcare facility-onset *Clostridioides difficile* infections decreased between cohorts.

β-Lactam vs. Alternative Therapy Use per Patients by Calendar Quarter

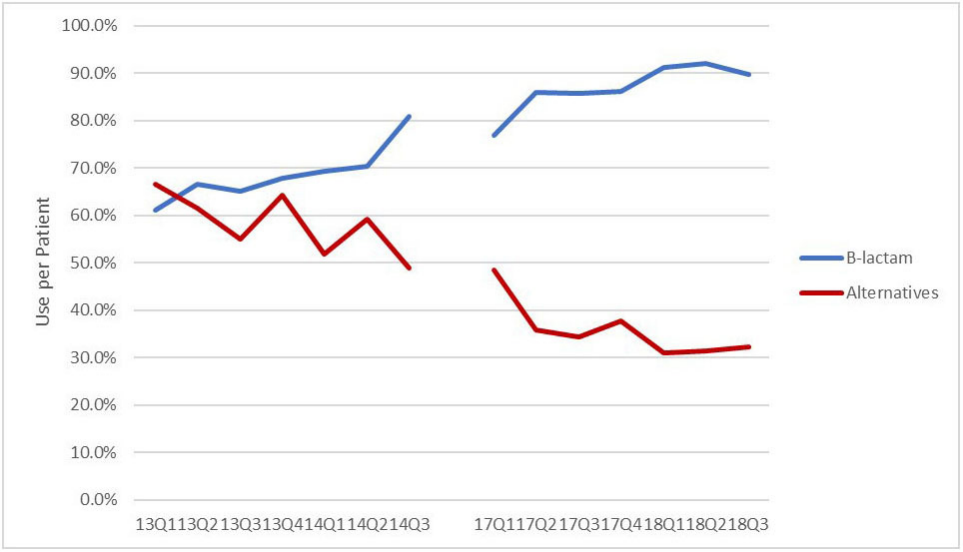

**Conclusion:**

Implementation of a β-lactam side-chain-based cross-reactivity chart significantly increased the utilization of β-lactams in patients with pneumonia without increasing HSRs.

**Disclosures:**

**All Authors**: No reported disclosures

